# Supplementary material for: Larval diapause slows adult epigenetic aging in an insect model, Nasonia vitripennis
Source: Proc Natl Acad Sci U S A. 2025 Jul 28;122(31):e2513020122. doi: 10.1073/pnas.2513020122 (PMC12337301; doi:10.1073/pnas.2513020122)
Supplement: Supplementary file 1 — Appendix 01 (PDF) [file pnas.2513020122.sapp.pdf]

Supporting Information for

Larval diapause slows adult epigenetic ageing in an insect model, *Nasonia vitripennis*

Erin B. Foley, Christian L. Thomas, Charalambos P. Kyriacou & Eamonn B. Mallon

Eamonn B. Mallon.
E-mail: ebm3@le.ac.uk

This PDF file includes:

Supporting text
Legends for Dataset S1 to S5
SI References

Other supporting materials for this manuscript include the following:

Datasets S1 to S5

Supporting Information Text

Methods

Rearing. *Nasonia vitripennis* used in this study were from the Leicester strain, a laboratory colony maintained at the University of Leicester for over nine years. This strain derives from AsymC, originally isolated in 1989 and subsequently cured of *Wolbachia* via heat shock treatment (1, 2).

To generate diapaused offspring, virgin females were housed at 20°C under a 8:16 h light:dark photoperiod. Host pupae were collected from Day 10 post-oviposition onwards to ensure recovery of diapaused larvae. These larvae developed to the fourth instar before being transferred to continuous darkness at 4°C for three months. After the diapause period, larvae were returned to standard rearing conditions and allowed to complete development to adulthood.

Lifespan Experiments. Diapaused and non-diapaused virgin male *Nasonia* were collected in batches within 24 hours of adult emergence (Day 0) and housed individually in plastic tubes. All individuals were maintained at 25°C and 40% relative humidity under a 12:12 h light:dark cycle. Each wasp received a daily feeding of 20 µL of 20% sucrose solution on filter paper, and mortality was recorded daily. Lifespan was defined as the number of days from adult emergence to death.

Kaplan–Meier survival analyses were performed using the survival package (v3.7) (3) and visualized with the survminer package (v0.4.9) (4). Cox proportional hazards models were fitted using the survival package. All analyses were conducted in R v4.4.1 (5).

Lifespan data were used to select sampling time points for whole-genome bisulfite sequencing (WGBS). Diapaused and non-diapaused virgin males were sampled at Days 6, 12, 18, 24, and 30, always at the same time of day. Upon collection, individuals were snap-frozen in liquid nitrogen and stored at –80°C until DNA extraction.

DNA Extraction. DNA was extracted using an adapted protocol based on the AllPrep® DNA/RNA Micro Kit (Qiagen). Each sample consisted of ten whole-body adult virgin male *Nasonia*. Four biological replicates were processed per time point (Days 6, 12, 18, 24, and 30).

DNA quality and concentration were assessed using a NanoDrop 2000 spectrophotometer (Thermo Fisher Scientific), a Qubit™ dsDNA BR Assay Kit (Thermo Fisher Scientific), and electrophoresis on a 1% agarose gel run for 40 minutes at 100 V. Samples were sent to Novogene (Beijing) for whole-genome bisulfite sequencing (WGBS). A 1% unmethylated lambda DNA spike-in was included in each sample to assess bisulfite conversion efficiency.

Whole-Genome Bisulfite Sequencing and Bioinformatic Processing. Raw sequencing data were provided in FASTQ format (Sequence Read Archive (SRA): PRJNA1275944). For samples sequenced across multiple lanes, read files were concatenated using the Unix `cat` command. Read quality was assessed using FastQC v0.12.1 (6).

A custom Snakemake pipeline (Snakemake v7.32.4; Python v3.12.1) (7) was used for preprocessing. Adapter sequences were trimmed and the first 10 bases removed from each read using Cutadapt v4.4 (8). Paired-end reads were aligned to the *Nasonia vitripennis* reference genome (Nvit_PSR1.1) (9) using Bowtie2 v2.5.1 with default parameters (10). Reads were also aligned to the unmethylated lambda genome (RefSeq accession: GCF_000840245.1) to assess bisulfite conversion efficiency.

Aligned reads were deduplicated and cytosine methylation calls extracted using Bismark v0.22.3 (11). Strand ambiguity was resolved using the `coverage2cytosine` utility in Bismark to generate destranded coverage files, which were used for downstream analyses.

Methylation Analysis. All downstream analyses were performed in R v4.4.1 (5). Destranded CpG coverage files were imported into the *MethylKit* package v1.30.0 (12) using the `methRead()` function. CpG sites with less than 10× coverage or coverage above the 99th percentile were filtered out. A binomial test was applied to each sample using the lambda genome conversion rate as the null probability of success, with a false discovery rate (FDR) threshold of $p < 0.05$ (SI Dataset S5). Only CpG sites showing significant methylation in at least one sample were retained. Percentage methylation at each site was calculated using the `percMethylation()` function.

Differential Methylation Analysis. Differential methylation analysis was performed using the *DSS* package v2.52.0 (13), which models methylation proportions using a beta-binomial generalized linear model (GLM) with an arcsine link function. A design matrix was constructed incorporating time point and treatment (diapause vs. non-diapause) as experimental factors. Linear models were fitted using the `DMLfit()` function to evaluate main effects and interactions. Differentially methylated loci (DMLs) and regions (DMRs) were identified accordingly.

Genomic features were assigned to CpG sites using a custom annotation file (GFF format) generated by Dr. Hollie Marshall using AGAT v0.10.0 (14).

Epigenetic Clock Construction. An elastic net regression model was trained to predict chronological age from DNA methylation levels across CpG sites, using data from virgin male *Nasonia vitripennis* sampled at five time points (Days 6, 12, 18, 24, and 30). Methylation data (percentage values) were filtered to retain CpG sites previously identified as differentially methylated across time using *DSS* (13).

To enhance model sparsity and robustness, CpG sites were pre-selected based on univariate Pearson correlation with age in the non-diapaused (control) group. Sites with absolute correlation ≥ 0.3 and uncorrected p-value ≤ 0.05 were retained. CpG features were then centered and scaled.

The final model was implemented using the `glmnet` algorithm with elastic net regularization ($\alpha = 0.5$), as part of the *caret* framework (15). Ten-fold cross-validation with three repeats was used for model tuning and performance estimation. The treatment group (diapause vs. control) was included as a categorical predictor, encoded as a dummy variable, and entered alongside the CpG methylation features. The optimal model was selected based on minimum root mean squared error (RMSE). Predictions were generated for all samples, and model performance was assessed using RMSE and R^2, stratified by treatment group.

Post-hoc analysis of predicted age trajectories was conducted using linear models with interaction terms, and estimated marginal means were compared between treatment groups at multiple time points using the *emmeans* package (16).

Calculation linking epigenetic age to mortality hazard. To assess whether the observed 2.7-day difference in epigenetic age at day 30 could plausibly explain the 65% reduction in mortality hazard observed in diapaused individuals (Diapause HR = 0.35), we used a simplified proportional hazards model in which mortality risk increases exponentially with epigenetic age:

$$h(t) = h_0(t) \cdot \exp(\beta \cdot \text{EpigeneticAge})$$

Under this model, the diapause hazard ratio associated with a difference in epigenetic age, Δ, is:

$$\text{HR}_{\text{diapause}} = \exp(\beta \cdot \Delta)$$

Rearranging, we solve for β:

$$\beta = \frac{\log(\text{HR}_{\text{diapause}})}{\Delta} = \frac{\log(0.35)}{-2.7} \approx 0.389$$

The corresponding hazard ratio per one additional day of epigenetic age is then:

$$\text{HR}_{\text{day}} = \exp(0.389) \approx 1.475$$

This implies that if epigenetic age were the sole mediator of the survival benefit conferred by diapause, each one-day increase in epigenetic age would be associated with a 47.5% increase in mortality hazard.

Gene Ontology Enrichment Analysis. Gene Ontology (GO) enrichment analysis was performed to assess the functional significance of genes associated with CpG sites included in the final epigenetic clock model. The 27 clock CpGs (SI Dataset S4) were mapped to nearby genes, which were then tested for enrichment against a background set comprising all genes associated with differentially methylated loci across time (SI Dataset S1). GO annotations were derived from *Nasonia vitripennis* and formatted for compatibility with *GOstats* (17).

The gene list was tested for overrepresentation of GO terms in the Biological Process (BP), Cellular Component (CC), and Molecular Function (MF) ontologies using a conditional hypergeometric test. Analyses were performed using the *GOstats* and *GSEABase* packages. For each ontology, both over- and under-representation were tested, and GO terms with an adjusted FDR of < 0.05 (Benjamini-Hochberg) were considered significant.

To visualise and summarise redundant GO terms, semantic similarity clustering was performed using the *rrvgo* package (18). Pairwise GO term similarities were calculated using the *org.Dm.eg.db* annotation database and the "Rel" semantic similarity measure. Representative GO terms were identified by reducing the similarity matrix with a similarity threshold of 0.7. Enriched terms were visualised using treemaps, heatmaps, and scatter plots. Final results were exported in tabular form (SI Dataset S5).

All scripts used are available at https://tinyurl.com/5n6vcvsk

SI Dataset S1 (SI_Datasets.xlsx/S1_Methylated_CpGs)

CpG sites classified as methylated using a binomial test with the lambda genome conversion rate as the null probability of success. Sites passing a false discovery rate (FDR) threshold of $p < 0.05$ and significantly methylated in at least one sample were retained.

SI Dataset S2 (SI_Datasets.xlsx/S2_Age_DMPs)

7,950 CpG sites showing significant age-associated differential methylation.

SI Dataset S3 (SI_Datasets.xlsx/S3_Age_Correlated)

289 age-associated CpG sites with strong correlation to chronological age (Pearson's $|r| \geq 0.3$, uncorrected $p \leq 0.05$).

SI Dataset S4 (SI_Datasets.xlsx/S4_Clock_Coefficients)

Elastic net model coefficients for the 27 CpG sites and intercept used to estimate epigenetic age.

SI Dataset S5 (SI_Datasets.xlsx/S5_GO_terms)

Enriched gene ontology terms associated with the 27 CpG sites comprising the epigenetic clock.

References

1. JH Werren, DW Loehlin, The parasitoid wasp Nasonia: an emerging model system with haploid male genetics. *Cold Spring Harb. Protoc.* **2009**, pdb–emo134 (2009) Publisher: Cold Spring Harbor Laboratory Press.
2. DC Darling, JH Werren, Biosystematics of Nasonia (Hymenoptera: Pteromalidae): two new species reared from birds' nests in North America. *Annals Entomol. Soc. Am.* **83**, 352–370 (1990) Publisher: Oxford University Press Oxford, UK.
3. Terry M. Therneau, Patricia M. Grambsch, *Modeling Survival Data: Extending the Cox Model.* (Springer, New York), (2000).
4. A Kassambara, M Kosinski, P Biecek, *survminer: Drawing Survival Curves using 'ggplot2'.* (2021).
5. R Core Team, *R: A Language and Environment for Statistical Computing.* (R Foundation for Statistical Computing, Vienna, Austria), (2024).
6. S Andrews, et al., FastQC (2012) Place: Babraham, UK Published: Babraham Institute.
7. J Köster, S Rahmann, Snakemake—a scalable bioinformatics workflow engine. *Bioinformatics* **28**, 2520–2522 (2012) _eprint: https://academic.oup.com/bioinformatics/article-pdf/28/19/2520/48879301/bioinformatics_28_19_2520.pdf.
8. M Martin, Cutadapt removes adapter sequences from high-throughput sequencing reads. *EMBnet.journal* **17**, 10–12 (2011).
9. E Dalla Benetta, et al., Genome elimination mediated by gene expression from a selfish chromosome. *Sci. Adv.* **6**, eaaz9808 (2020) Publisher: American Association for the Advancement of Science.
10. B Langmead, SL Salzberg, Fast gapped-read alignment with Bowtie 2. *Nat. methods* **9**, 357–359 (2012) Publisher: Nature Publishing Group.
11. F Krueger, SR Andrews, Bismark: a flexible aligner and methylation caller for Bisulfite-Seq applications. *bioinformatics* **27**, 1571–1572 (2011) Publisher: Oxford University Press.
12. A Akalin, et al., methylKit: a comprehensive R package for the analysis of genome-wide DNA methylation profiles. *Genome biology* **13**, 1–9 (2012) Publisher: Springer.
13. H Feng, H Wu, Differential methylation analysis for bisulfite sequencing using DSS. *Quant. Biol.* **7**, 327–334 (2019) Publisher: Springer.
14. J Dainat, AGAT: Another Gff Analysis Toolkit to handle annotations in any GTF/GFF format.(Version v0. 7.0). *Zendo. doi* **10** (2023).
15. M Kuhn, Building Predictive Models in R Using the caret Package. *J. Stat. Softw.* **28**, 1–26 (2008).
16. RV Lenth, emmeans: Estimated marginal means, aka least-squares means, manual (2022).
17. S Falcon, R Gentleman, Using GOstats to test gene lists for GO term association. *Bioinformatics* **23**, 257–258 (2007) Publisher: Oxford University Press.
18. S Sayols, rrvgo: a Bioconductor package for interpreting lists of Gene Ontology terms. *microPublication Biol.* (2023) Publisher: Caltech Library.
